# Antibiotic Potential and Biophysical Characterization of Amphipathic β-Stranded [XZ]_n_ Peptides With Alternating Cationic and Hydrophobic Residues

**DOI:** 10.3389/fmedt.2021.622096

**Published:** 2021-02-04

**Authors:** Erik Strandberg, Parvesh Wadhwani, Anne S. Ulrich

**Affiliations:** ^1^Karlsruhe Institute of Technology, Institute of Biological Interfaces (IBG-2), Karlsruhe, Germany; ^2^Karlsruhe Institute of Technology, Institute of Organic Chemistry, Karlsruhe, Germany

**Keywords:** linear β-stranded antimicrobial peptides, cationic membrane-active peptides, peptides with alternating cationic and hydrophobic residues, biophysical studies of peptides in membranes, circular dichroism spectroscopy, fluorescence spectroscopy, peptide folding, peptide aggregation

## Abstract

Cationic membrane-active peptides are considered to be promising candidates for antibiotic treatment. Many natural and artificial sequences show an antimicrobial activity when they are able to take on an amphipathic fold upon membrane binding, which in turn perturbs the integrity of the lipid bilayer. Most known structures are α-helices and β-hairpins, but also cyclic knots and other irregular conformations are known. Linear β-stranded antimicrobial peptides are not so common in nature, but numerous model sequences have been designed. Interestingly, many of them tend to be highly membranolytic, but also have a significant tendency to self-assemble into β-sheets by hydrogen-bonding. In this minireview we examine the literature on such amphipathic peptides consisting of simple repetitive sequences of alternating cationic and hydrophobic residues, and discuss their advantages and disadvantages. Their interactions with lipids have been characterized with a number of biophysical techniques—especially circular dichroism, fluorescence, and infrared—in order to determine their secondary structure, membrane binding, aggregation tendency, and ability to permeabilize vesicles. Their activities against bacteria, biofilms, erythrocytes, and human cells have also been studied using biological assays. In line with the main scope of this Special Issue, we attempt to correlate the biophysical results with the biological data, and in particular we discuss which properties (length, charge, aggregation tendency, etc.) of these simple model peptides are most relevant for their biological function. The overview presented here offers ideas for future experiments, and also suggests a few design rules for promising β-stranded peptides to develop efficient antimicrobial agents.

## Introduction

**Figure 1:**
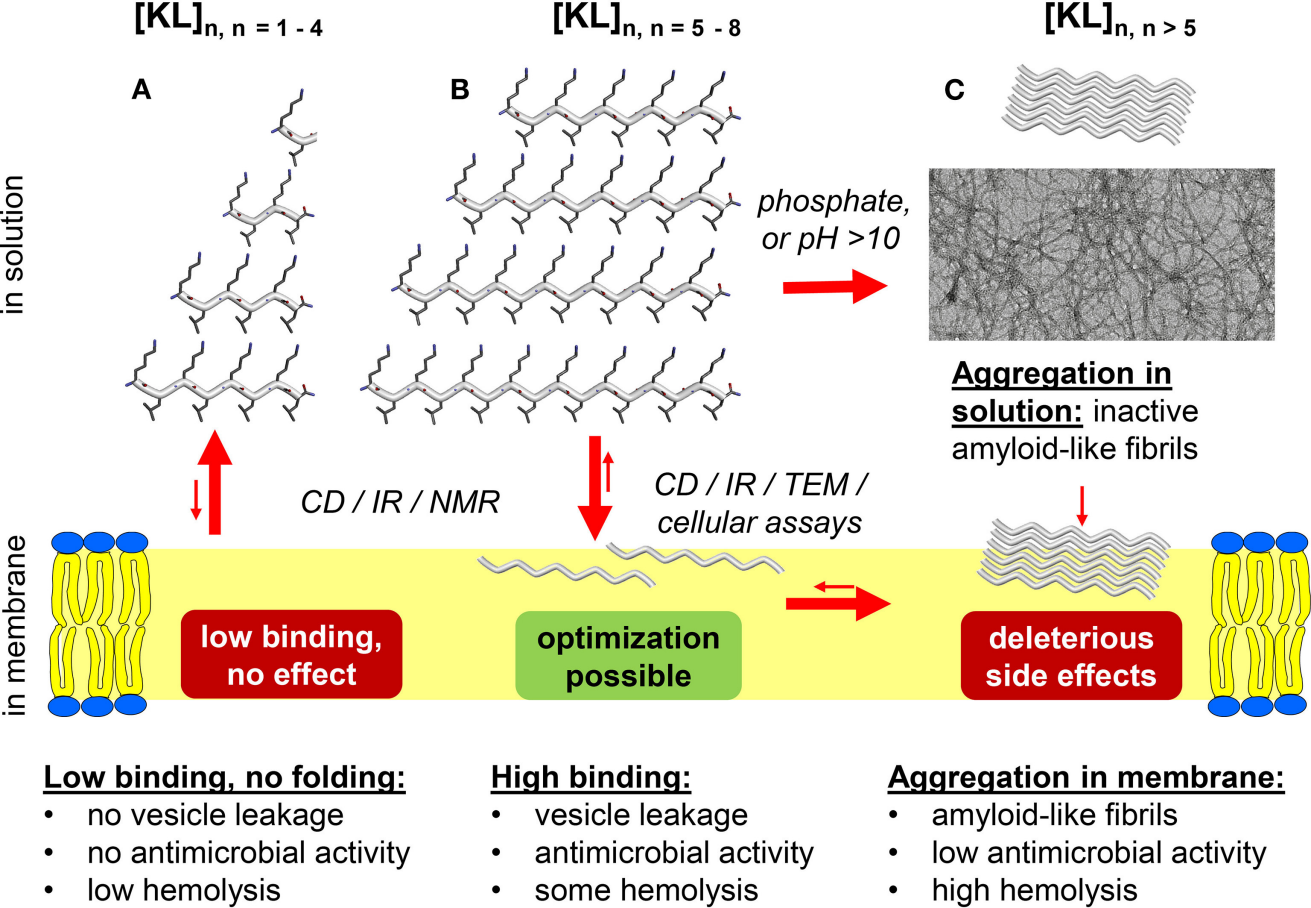
Overview of the length-dependent membrane perturbing activity of peptides composed of alternating cationic and hydrophobic residues. General XZ sequences are here illustrated by [KL]_n_, which have been studied extensively by biophysical and spectroscopic methods, as well as electron microscopy and microbiology. **(A)** Low binding, no effect: Very short KL peptides with up to 8 residues show a very low ability to bind or to fold in the membrane, and thus have essentially no antimicrobial activity. They do not self-assemble in the lipid bilayer, so they do not show much membrane toxicity in form of hemolysis either. **(B)** Optimization possibility: Medium-length KL peptides show higher binding and folding, hence they induce good membrane activity in the form of antimicrobial action and vesicle leakage. Based on length and kinetics of aggregation, they are able to self-assemble in the membrane into amyloid-like fibrils and therefore demonstrate moderate hemolytic side effects. **(C)** Side effects: KL peptides longer than 10 residues display a pronounced phosphate-dependent pre-aggregation already in solution, which causes extensive loss of the active molecules in form of toxic aggregates, before they can even bind to the membrane. This loss of material causes a lowering of the desired antimicrobial membrane activity, while at the same time the strong (pre-)aggregation correlates with increased hemolysis.

Cationic, amphipathic membrane-active peptides are considered to be promising new candidates for antibiotic treatment (1, 2). The simplest possible such peptides are made up from an alternate repeat sequence of cationic and hydrophobic amino acids, which can be expected to form amphipathic β-strands in a membrane (see Figure 1), with a fully extended backbone according to the Ramachandran plot (as in a β-sheet, but considering only a single strand here). All cationic residues will be aligned on one face and point toward the water, while the hydrophobic residues on the opposite side point into the hydrophobic interior of the membrane. Such amphipathic structures are usually membrane-active, and several peptides of this type have indeed shown antimicrobial activity.

A lot of interest has been shown in α-helical AMPs, but there is a lack of review articles covering amphipathic β-stranded peptides, and we therefore here try to summarize all relevant publications from 1981 to date about simple repetitive sequences able to form amphipathic β-strands (see Table 1).

**Table 1:** List of peptide sequences and which methods have been used to study them.

**Peptides^**a**^**	**Sequences**	**Methods^**b**^**	**References**
KI	[IK]_4_-NH_2_	BFA, CD, EM, FL, HA, MIC	(3)
KL	poly-[LK]	CD, IR	(4)
KL	L[KL]_3_	CD, IR, ML	(5)
KL	Acetyl-[KL]_9_-NH_2_	HA, MIC, RT	(6)
KL	[KL]_n_, *n* = 8,10	CD, IR, VCD	(7)
KL	Dansyl-[KL]_4_K-NH_2_, Dansyl-[KL]_5_K-NH_2_, Dansyl-[KL]_6_K-NH_2_, Dansyl-[KL]_7_K-NH_2_	BA, FL, HA, IR, LA, ML, RT	(8)
KL	Dansyl-[KL]_4_K-NH_2_, Dansyl-[KL]_5_K-NH_2_, Dansyl-[KL]_7_K-NH_2_	CA, MIC	(9)
KL	[LK]_n_, *n* = 2,5,7,8,9,10,11,12,18,24	AF, HA, MIC	(10)
KL	[KL]_4_K, [KL]_7_K	CD, RS	(11)
KL	[KL]_3_-NH_2_, [KL]_5_-NH_2_, [KL]_7_-NH_2_, [KL]_9_-NH_2_	BA, CD, EM, HA, LA, MIC	(12)
KL	[KL]_n_, *n* = 3,4,5,6,7,8,9,10,11,12,13; [LK]_5_; L[KL]_n_, *n* = 4,5,6,7; [KL]_n_K, *n* = 4,5,6,7 (all –NH_2_)	CD, HA, LA, MIC, NMR, OCD	(13)
KV	Ac-[KV]_n_-NHCH_3_, *n* = 2,3,4	CD, FL, LA, MIC	(14)
KV	C_16_-[VK]_4_	AF, CA, CD, EM, ITC, MIC	(15)
KW	[KW]_3_, [WK]_3_	AF, BA, CD, HA, MIC, LA	(16)
KW	[KW]_2_-NH_2_, [KW]_3_-NH_2_, [KW]_4_-NH_2_, [KW]_5_-NH_2_	AF, CV	(17)
RI	[IR]_4_-NH_2_	BFA, CD, EM, FL, HA, MIC	(3)
RL	[LR]_n_, *n* = 7,9,11	AF, HA, MIC	(10)
RW	[RW]_2_R-NH_2_, W[RW]_2_-NH_2_, [RW]_3_-NH_2_	HA, MIC	(18)
RW	RWR-NH_2_, WRW-NH_2_, [WR]_2_-NH_2_, R[WR]_2_-NH_2_, [WR]_2_W-NH_2_, [RW]_3_-NH_2_, [WR]_3_-NH_2_	MIC	(19)
RW	RW-NH_2_, [RW]_2_-NH_2_, [RW]_3_-NH_2_, [RW]_4_-NH_2_, [RW]_5_-NH_2_	BA, CD, FL, HA, MIC	(20)
RW	[RW]_2_-NH_2_, [RW]_3_-NH_2_, [RW]_4_-NH_2_, [RW]_5_-NH_2_	AF, CV	(17)
RW	[RW]_3_-NH_2_, lipidation	CV, HA, MIC, RT	(21)
X_1_Z_1_X_2_Z_2_	[VRVK]_2_-NH_2_, [VRVK]_3_-NH_2_, [IRIK]_2_-NH_2_, [IRIK]_3_-NH_2_, [IRVK]_2_-NH_2_, [IRVK]_3_-NH_2_, [FRFK]_2_-NH_2_, [WRWK]_2_-NH_2_	BFA, CD, EM, FL, HA, MIC	(3)

a *The repetitive sequence is given; charged residues are always stated first (i.e., [KL]_n_ and [LK]_n_ peptides are all listed under KL)*.

b *Abbreviations: AF, antifungal assay; BA, binding assay; BFA, biofilm assay; CA, cell assays; CD, circular dichroism spectroscopy; CV, cell viability assay; EM, electron microscopy; FL, fluorescence spectroscopy; HA, hemolysis assay; IR, infrared spectroscopy; ITC, isothermal titration calorimetry; LA, leakage assay; MIC, minimum inhibitory concentration assay; ML, monolayer studies; NMR, nuclear magnetic resonance; OCD, oriented CD; RS, Raman spectroscopy; RT, HPLC retention time; VCD, vibrational CD*.

The peptides covered here have repeating alternating sequences of cationic and hydrophobic amino acids, i.e., of the type [XZ]_n_ or [ZX]_n_, where X is a cationic and Z a hydrophobic amino acid. Using one-letter codes for natural amino acids, X can be K (lysine) or R (arginine), and Z can be V (valine), L (leucine), I (isoleucine), W (tryptophan) or F (phenylalanine). This gives ten XZ combinations, and most but not all of them have been studied (see Table 1). KL peptides have seen the largest interest, and also RL peptides have been examined. KI and RI peptides were studied as well, but only with a fixed length of eight amino acids. KW and RW peptides with different lengths have been investigated by several groups. We also found studies of KV but not of RV peptides. KF and RF peptides seem to have been ignored so far.

The main questions this minireview will try to answer are: (**Q1**) Do such repetitive sequences bind to membranes and form β-strands? (**Q2**) Do they show high antimicrobial activity? (**Q3**) Are the peptides selective, in terms of high antimicrobial activity vs. low hemolytic side effects? (**Q4**) Is there an optimal length of these peptides? (**Q5**) What is their mechanism of membrane permeabilization?

These questions are investigated using different methods. Binding, secondary structure and leakage are studied with biophysical methods in vesicles or monolayers, whereas antimicrobial and hemolytic activities are determined using biological assays. A main theme in this review is therefore to combine results from vesicle and cell studies.

## Peptide Binding to Membranes

Fluorescence methods have been used to study peptide binding to membranes. Trp fluorescence is sensitive to the environment and it is therefore a preferred method to use for peptides containing Trp. [WK]_3_ and [KW]_3_ were shown to bind to anionic lipid vesicles using this method (16). RW peptides with 2–10 amino acids bind to anionic phosphatidylglycerol (PG) vesicles (20). A series of KL peptides with different length were labeled at the N-termini with NBD, a fluorophore whose intensity is sensitive to the environment. Amongst these KL peptides, those with 6–18 amino acids had a high affinity for vesicles composed of neutral (zwitterionic) POPC and negatively charged POPG lipids (POPC/POPG, 1/1 mol/mol), and the longer ones were found to bind most strongly (12). In another study of dansyl-labeled KL peptides, peptides with 9–15 amino acids were binding to egg-PC/cholesterol (10/1) membranes with a higher lipid affinity for longer peptides (8).

These results are mostly quantitative, and only for KL peptides were binding constants calculated (8, 12). But it is obvious to conclude that the cationic XZ peptides experience a long-range electrostatic attraction to negatively charged membranes, and hydrophobic interactions can drive the binding further. In the case of RW, even very short dipeptides bind (20).

## Assuming a β-Stranded Conformation in Membranes

We now consider question (**Q1**) above, whether the amphipathic XZ peptides indeed take on a β-stranded conformation when bound to membranes. Circular dichroism spectroscopy (CD) is the method of choice to study the secondary structure of peptides (22, 23), but cannot define the degree of assembly or oligomerization. Infrared spectroscopy (IR) can also be used to determine the local secondary structure (24), provided that the material is free from trifluoroacetic acid (25). It is not always possible with these methods to discriminate between monomeric β-strands, oligomeric β-sheets or long amyloid-like cross-β-sheet fibrils, so we will simply refer to a β-stranded conformation. X-ray diffraction and/or transmission electron microscopy (TEM) would be needed to characterize any assembly on the supramolecular scale. The published results from CD and IR studies on XZ peptides are summarized in Supplementary Table 1, and are discussed below.

KL peptides with 6–26 amino acids were studied with CD in pure water and shown to bind to POPC/POPG vesicles. Peptides consisting of eight or more amino acids exhibited distinct β-stranded line shapes (13). [IR]_4_ and [IK]_4_ are unstructured in pure water, but show a β-stranded conformation in the presence of SDS micelles (no buffer was used) (3). [KV]_4_–but not [KV]_2_ and [KV]_3_–binds to anionic vesicles in HEPES buffer and becomes β-stranded (14).

Most AMPs depend on a membrane environment to fold into their active amphipathic structure; in contrast, some XZ peptides have been found to do so in solution without a membrane, especially in the presence of phosphate.

CD spectra of [KW]_3_ and [WK]_3_ showed line shapes similar to α-helical conformations in 10 mM phosphate buffer and in the presence of neutral vesicles, but they bind to anionic vesicles and turn β-stranded (16). Monolayer studies were done for KL peptides at the water-air interface, with and without DMPC lipids. Peptides with 9–15 amino acids were found to give characteristic antiparallel β-sheet IR line shapes under all conditions, with peptides oriented flat on the membrane surface (8). Poly-LK ([LK]_n_, where n is not exactly known, but is a large number) was shown with CD and IR to form antiparallel β-sheets *per se* in aqueous solution in absence of lipids (4). A later study using both electronic and vibrational CD, combined with IR, also showed that [KL]_8_ and [KL]_10_ form antiparallel β-sheets in D_2_O at high peptide concentration (7). The aggregation of KL peptides into β-sheet amyloid-like fibrils (as was confirmed by TEM) in solution was found to be enhanced by buffers containing phosphate ions, and the speed of folding increases with phosphate concentration and peptide length (12). The short sequence of [KL]_3_ did not fold, but aggregation was very fast for KL peptides with 14 or more amino acids (12). RW peptides with 4–10 amino acids showed signs of β-stranded CD signals in 20 mM phosphate buffer (20).

This means that in any studies using membranes—model bilayers as well as native cells—in phosphate buffer (like the RW and KL peptides above), it is not clear whether the observed folding occurs in the lipid bilayer or the peptide have pre-aggregated already in solution. As it was seen in the previous section that these cationic peptides tend to bind strongly to anionic lipids, it seems reasonable to assume that at least some of the peptides are located in the membrane in a β-stranded conformation.

Furthermore, a minimum length seems to be required for β-strand formation in the membrane (in absence of phosphate). KL peptides (13), and KV peptides (14) needed at least eight amino acids, as shorter peptides did not fold at all. For KI and RI peptides, a length of eight amino acids is also enough (shorter ones were not tested) (3). For RW peptides already tetrapeptides formed β-strands (20), though it is not clear if this would also be the case without any phosphate ions present in the medium.

## Membrane Damage, Vesicle Leakage

After establishing that the XZ peptides bind to anionic membranes in β-stranded conformations above a certain length, the question arises whether the peptides perturb the lipid bilayer and induce damage. This can be tested using fluorescence-based vesicle leakage assays. Several assays are available, and several types of XZ peptides have been tested:

- KL peptides were tested using an ANTS/DPX assay (12, 13). In POPC/POPG (1/2) vesicles, peptides with at least 10 amino acids showed >80% leakage, while shorter peptides were mostly inactive at a peptide-to-lipid molar ratio (P/L) of 1/20 (12).
- KV peptides were tested using a carboxyfluorescein assay in DPPC/DPPG (3/1) at P/L=1/10 (14). At 45°C, when the lipids were in a liquid crystalline phase, [KV]_2_ gave no leakage, with [KV]_3_ around 20% leakage was observed, and with [KV]_4_ up to 50% leakage was found.
- [KW]_3_ and [WK]_3_ were tested using a calcein leakage assay (16). Leakage was strongly concentration dependent and also lipid-dependent. In anionic EYPC/EYPG (7/3) both peptides gave over 50% leakage at P/L = 1/10.

It is hard to compare these results on different peptides, since different lipids, buffers and dyes were used, and peptide concentrations varied. Only for the KL series, a wide range of peptide lengths has been systematically examined. It seems that KW and KV peptides are active already when they are at least six and eight amino acids long, respectively, whereas KL peptides needs to be at least 10 amino acids long to cause leakage. However, the minimum length required for the activity might vary and depend strongly on the experimental conditions.

At least for the KL and KV peptides, there is a correlation between folding into a β-stranded conformation and vesicle leakage activity. The short [KL]_3_ binds to the membrane, but does not form β-strands and does not induce leakage (Figure 1A). [KL]_5_ binds stronger than [KL]_3_, forms β-strands (Figure 1B) and can also induce leakage (12). The longer KL peptides have a medium to high tendency to aggregate already in aqueous conditions as β-sheet fibrils (Figure 1C), especially in the presence of phosphate ions. KV peptides with eight amino acids formed β-strands and also induced leakage, whereas the shorter analogs did not fold into β-strands and gave no or low leakage (14).

## Antimicrobial Activity

Comparing the results of antimicrobial assays between different studies in a quantitative manner is virtually impossible, because different experimental conditions and different bacteria have been used. Here we try to establish whether a peptide is active against any bacteria or not. MIC (minimum inhibitory concentration) values <10 μg/mL or μM, we will call “high activity”; MIC values of 10–100 μg/mL or μM, “medium activity”; and MIC values >100 μg/mL or μM, “low activity.” If no activity was found at the highest tested concentration, we will call it “no activity.” Results of studies of antimicrobial activity of XZ peptides are discussed below. More details can be found in Supplementary Table 2, where also the microbes used are specified.

- KL peptides with 6–26 amino acids showed a length dependent activity in MIC tests against four different bacterial strains, with low activity for 6-mers, medium activity for peptides with eight amino acids, high activity for intermediate lengths (9–15 amino acids), and medium activity for longer peptides (13). The activity was slightly better against Gram-positive than Gram-negative bacteria, but usually not by more than a factor of two. The observed MIC values strongly depended on how the assay was performed, e.g., MIC values increased up to a factor 32 when peptides were exposed to phosphate buffer (12), illustrating how ambiguous it is to compare results from different studies.
- KL peptides (labeled with dansyl at the N-terminus) with nine amino acids showed high activity against some Gram-positive and Gram-negative strains, whereas peptides with 11 or 15 amino acids only showed medium activity (9).
- LK peptides with 14–24 amino acids all showed a similar, low to medium activity against Gram-positive and Gram-negative bacteria (10). LR peptides with 14 or 18 amino acids were compared in the same study. [LR]_7_ had a similar activity as [LK]_7_, but [LR]_9_ and [LR]_11_ had almost no activity. The LK and LR peptides were also tested for anti-fungal activity, and only the peptides with 14 amino acids showed a high activity; longer ones showed medium or low activity and shorter ones were not tested (10).
- [IR]_4_ had a high activity against Gram-positive and Gram-negative bacteria and medium activity against *C. albicans*; [IK]_4_ showed high activity against Gram-positive bacteria and *C. albicans*, and medium activity against Gram-negative bacteria (3). The difference in MIC between [IR]_4_ and [IK]_4_ could be as large as a factor of 8.
- KV peptides with 4–8 amino acids did not show any antimicrobial activity (up to 100 μg/ml peptide concentration) (14). [KV]_4_ with a hexadecanoic acid chain attached to the N-terminus also did not show any activity (up to 32 μg/ml peptide) against 11 tested microbes (15). This indicates that membrane affinity (binding) and folding into an amphipathic β-stranded conformation is not sufficient for antimicrobial activity.
- [KW]_3_ and [WK]_3_ had medium or low activity against many bacteria, and somewhat higher activity against Gram-negative than Gram-positive bacteria. [KW]_3_ usually had a lower MIC value than [WK]_3_, by a factor of 2 (16).
- [KW]_2_ and [RW]_2_ showed no antifungal activity, but [KW]_3_, [RW]_3_, and [RW]_4_ had a medium and [KW]_4_, [KW]_5_, and [RW]_5_ had a high activity against *F. solani* and *F. oxysporum* (17).
- RW peptides have been examined in several studies. [RW] and [RW]_2_ had no activity, [RW]_3_ medium activity, [RW]_4_ and [RW]_5_ high activity against *E. coli* and *S. aureus* (20). Another study found that [RW]_3_ had high activity while W[RW]_2_ and [RW]_2_R showed medium activity against three tested bacteria (18). The same group later tested further peptides with 3–6 amino acids, and found that peptides with 2 or 3 amino acids had low or no activity, W[RW]_2_ had medium activity and was more active than [RW]_2_R, and [RW]_3_ and [WR]_3_ had a high activity (19). In another study, a Lys residue with an attached acyl chain was added to the N- or C-terminus of [RW]_3_, and the effect of different acyl chain lengths was investigated (21). [RW]_3_ itself had high activity against Gram-positive and medium activity against Gram-negative bacteria. In contrast to the KV peptide, here the activity improved when an acyl chain with 8–12 carbons was added (21).

From these results it can be concluded that (i) The activity of XZ peptides covers a broad spectrum; activity is similar against Gram-positive and Gram-negative bacteria, and some have also high activity against fungi. (ii) KV and RV peptides are much less active than the other combinations. (iii) XZ peptides need a minimum threshold length to be highly active, and from the studies mentioned 6–9 amino acids appear to be the threshold length. (iv) Activity depends on the sequence, e.g., Trp-containing peptides are active already with six amino acids, whereas Leu peptides needs at least nine amino acids. For IR and IK, only peptides with eight amino acids were tested and they had medium to high activity. For KW, peptides with six amino acids showed medium activity. Peptides containing Ile or Val have not been studied in sufficient detail to determine a threshold length. (v) For KL peptides, the minimum length needed for activity is similar to the minimum length needed for folding into a β-stranded conformation in membranes, and it is also similar to the threshold length needed for vesicle leakage (12, 13). (vi) KL peptides with more than 14 amino acids are less active (even when compared in a weight-by-weight manner) than the mid-length analogs. This intriguing observation was attributed to the enhanced tendency of the long peptides to pre-aggregate in solution, as illustrated in Figure 1C (12, 13). LK and LR peptides also seem to become less active when they get too long (10). The other XZ combinations have not been tested using long peptides with more than 10 amino acids, so it may be too early to conclude that they would also become less active when they get longer, but it seems very likely.

## Hemolysis

Comparing hemolysis values from different studies should be approached with caution, as human blood is not a standardized product, different protocols are used, and some studies present HC_50_ numbers (peptide concentration for 50% hemolysis) whereas others gives the percentage of hemolysis at a given peptide concentration. Nonetheless, this erythrocyte-based assay indicates whether a certain peptide show side-effects against human cells or not.

- In a study of KL peptides with 6–26 amino acids, hemolytic activity was low for peptides up to 10 amino acids (HC_50_ >40 μg/mL), but very high for 14 or more amino acids (HC_50_ ≤ 2 μg/mL) (13). In another study, acetyl-[KL]_9_-NH_2_ gave negligible hemolysis at 100 μg/ml (6).
- [IR]_4_ and [IK]_4_ gave only low hemolysis (HC_10_ >100 μg/mL) (3).
- [KW]_3_ and [WK]_3_ showed no hemolysis up to a concentration of 400 μM (16).
- RW with 2–10 amino acids showed overall low hemolysis (HC_50_ >75 μg/mL), with longer peptides being more active (20). [RW]_3_, W[RW]_2_, and [RW]_2_R showed no hemolysis up to 1,000 μg/ml (18). [RW]_3_ showed low hemolysis also in another study (<10% at 250 μg/ml), but it increased upon lipidation (21). In the latter study, the effect of RW peptides on cell lines was also studied. [RW]_3_ with a C_8_ acyl chain connected to the N- or C-terminus was toxic to cancer cell lines (IC_50_ ≈ 4 μM against MCF7 and HT29 cells), but less toxic to non-malignant fibroblast (GM5657) cells (IC_50_ ≈ 32 μM) (21).

Even if it hard to compare the hemolytic effects found in different studies, is seems safe to state that short peptides with up to 10 amino acids give only low hemolysis, independent of the exact sequence. We believe that this is due to their slow kinetics to aggregate in the membrane into toxic fibrils. KL peptides with more than 14 amino acids, which have a high tendency to aggregate, are on the other hand highly hemolytic. Other sequences have not been tested using peptides with more than 10 amino acids, but there is a clear trend toward higher activity for longer peptides, so it seems reasonable to assume that any XZ peptide would also be highly hemolytic once it is long enough. Note that this continual upwards trend in hemolysis differs from the corresponding antimicrobial effects, which become reduced again for the very long peptides.

## Selectivity

The selectivity of peptides can be determined by comparing MIC values and hemolysis. Only a few studies have investigated both MIC and hemolysis. We here try to compare MIC with HC_50_ and define high selectivity when the therapeutic index (TI = HC_50_/MIC) is at least 10.

For KL peptides, high selectivity was found for peptides with 8–10 amino acids, but for peptides with 14 or more amino acids, TI was below 1 (13). For RW peptides, one study found no hemolysis (18), which indicates high selectivity. Another study found TI > 10 for RW peptides with 4–10 amino acids (20). For [RW]_3_, high selectivity was found, also when acyl chains were attached to the N- or C-terminus (21). From these results we conclude that for short XZ peptides with up to 10 amino acids, selectivity is high.

## Longer Repetitive Sequences

Some longer repetitive sequences of the type [X_1_Z_1_X_2_Z_2_]_n_ have been examined, but no systematic overview of all combinations has been made. In one study, seven peptides of the type [Z_1_KZ_2_R]_n_ were compared to [IR]_4_ and [IK]_4_ (3). The results were similar as for the [XZ]_n_ peptides discussed above. All peptides gave β-stranded CD line shapes in SDS micelles. For hemolysis, two trends were observed: (i) longer peptides with the same repetitive sequence produced more hemolysis, which may be due to self-assembly as discussed above; and (ii) peptides containing V or F were less hemolytic than peptides containing I or W, which fits with the observation that more hydrophobic peptides are usually more hemolytic (26–28). MIC tests against four bacterial strains showed that no peptide performed the best in all cases. Overall, the authors found that [IRIK]_2_ and [IRVK]_3_ were the most promising peptides (3).

## Mechanism of Action

The basic assumption is that the antimicrobial activity of XZ peptides is due to membrane interactions. Many models have been proposed how AMPs can lead to membrane damage, and some indications to the mechanism of action can come from biophysical studies.

[KL]_n_K peptides were shown with IR to lie flat on the surface of an air-water interface, or a DMPC monolayer (8). [KL]_5_ and [KL]_7_ have been studied with solid-state ^19^F-NMR in several lipid bilayer systems, and were found to lie flat on the membrane surface for all lipid compositions, independent of peptide concentration (13). A transmembrane orientation could indicate that peptides form pores through the membrane, but this was not observed. Leakage of KL peptides could be correlated with peptide length (even at constant weight-to-weight), but there was no dependence on membrane thickness. A certain dependence on bilayer thickness would have been expected if the peptides were to assemble into a transmembrane pore, such as a β-barrel. All in all, there was no sign of pore formation, and the most likely mechanism was proposed to be a carpet mechanism, with peptides on the membrane surface destabilizing the membrane by lateral crowding (13). Other XZ peptides have not been studied with these structural methods, so it is not clear if they all use the same mechanism of action. Apart from membrane permeabilization, it is likely that these cationic peptides can also disturb membrane integrity by clustering of anionic lipids, as found for other cationic AMPs (29). It is also possible that the peptides can pass through the membrane and be active against internal targets in the cell.

## Balance Between Charged and Hydrophobic Residues

XZ peptides have repeating alternating sequences of cationic and hydrophobic amino acids. If the number of residues is odd, then there will be more cationic or more hydrophobic residues, and the effects of this shift in the balance between charge and hydrophobicity can be observed in some studies.

In two studies, [WR]_2_W showed a higher antimicrobial activity than R[WR]_2_ (18, 19). In another study, [RW]_3_ was a bit more active than K[RW]_3_ or [RW]_3_K (21). This seems to indicate that too much charge is reducing the antimicrobial activity, so that an overweight of hydrophobic residues may be advantageous. For KL peptides, it was found that [KL]_4_K had a slightly lower antimicrobial activity than L[KL]_4_, and [KL]_5_K was less active than L[KL]_5_. But on the other hand, [KL]_6_K was clearly more active than L[KL]_6_, and [KL]_7_K was much more active than L[KL]_7_ (13). Thus, it seems that the balance shifts for longer peptides, so that higher hydrophobicity gives lower activity. This can be explained by the increased tendency of longer peptides to form aggregates, which reduces the antimicrobial activity. When peptides are more hydrophobic, this aggregation tendency is increased. For the short peptides, on the other hand, aggregation propensity is low so aggregation is not an issue, and it is likely that short peptides with less hydrophobicity have a lower affinity for membranes and are therefore less active.

For peptides with an even number of residues, there are as many cationic as hydrophobic residues. Here, the balance between hydrophobicity and charge mainly depends on the nature of the hydrophobic residues. Studies of these peptides show that the hydrophobicity has to be high enough: Val seems not to be sufficiently hydrophobic, and KV peptides are not active up to a length of eight residues (14). Leu is more hydrophobic than Val and KL peptides with eight residues have medium activity (13). Trp is probably the most hydrophobic and interfacially active residue, and this can be the reason KW and RW peptides show high activity even with six residues (17, 19, 20). Also in this case, the behavior changes with peptide length. [VRVK]_2_ is much less active than [IRIK]_2_, but [VRVK]_3_ is more active than [IRIK]_3_ (3).

Another way of shifting the balance between charge and hydrophobicity is to add an acyl chain to the peptide, which was done for [RW]_3_ peptides (21). Adding a chain with 2 or 4 carbons did not improve the activity, but chains of 6–12 carbons improved MIC values. This fits with the observation above that for short peptides, additional hydrophobicity can improve activity.

Terminal groups can also modify the balance of charge and hydrophobicity. C-terminal amidation leads to a higher net positive charge and higher hydrophobicity. N-terminal acetylation leads to less positive charge and higher hydrophobicity. As can be seen in Table 1, some studies have used peptides with amidated or acetylated termini, but we have not found any studies where peptides with different terminal groups have been compared. From the results mentioned above, we predict that acetylation would be good for short but bad for long peptides. The effect of amidation is harder to predict, since both positive charge and hydrophobicity are increased.

More hydrophobic peptides are more hemolytic. For KL peptides, in all tested cases L[KL]_n_ peptides showed more hemolysis than [KL]_n_K (*n* = 4,5,6,7) (13). For Z_1_KZ_2_R peptides, the amount of hemolysis of peptides increased according to [VRVK]_2_ < [IRVK]_2_ < [IRIK]_2_ (3), which correlates with the hydrophobicity. For short peptides, where higher hydrophobicity led to higher antimicrobial effects, and hemolysis is low, increased hydrophobicity can be good for selectivity, but for long peptides lower hydrophobicity is preferable since this can both increase antimicrobial activity and reduce hemolysis.

## Therapeutic Potential

Before peptides can be used for therapy, many questions must be addressed. Are the peptides stable against proteases or will they be quickly degraded in the body? Will the pathogens develop resistance against the peptides? Will they be effective not only against bacteria *in vitro* but also *in vivo*? We have not found any studies of XZ peptides addressing these issues, so these are open questions where future studies would be needed.

## Conclusions

We now return to the questions posed in the introduction about amphipathic [XZ]_n_ sequences. (**Q1**) Do such repetitive sequences bind to membranes and form β-strands? **Answer**: Yes, if they are at least 6–10 amino acids long. (**Q2**) Do they show high antimicrobial activity? **Answer**: Yes, many of the tested sequences have shown a high activity (MIC < 10 μg/ml) against Gram-positive and Gram-negative bacteria, and against fungi. Peptides containing Val are less active. However, not all peptides are highly active against all bacteria, and different peptides showed the best activity against different bacteria, so testing is necessary to find the best candidate against a specific pathogen. (**Q3**) Are the peptides selective, in terms of high antimicrobial activity vs. low hemolytic side effects? **Answer**: Yes. All peptides with up to 10 amino acids show low or very low hemolysis, so if they are active against the relevant microbes then hemolysis seems not to be a problem. (**Q4**) Is there an optimal length of these peptides? **Answer**: Yes. Too short peptides do not bind and fold, and too long peptide aggregates and have reduced antimicrobial activity and increased their hemolytic activity, so there is an optimal length, which is different for different sequences, and seems to be related to the hydrophobicity of the Z residues. For KL peptides, the ideal length is 9–11 amino acids. For KW and RW peptides, 6–8 seems good. For the other sequences, not enough different lengths have been tested to answer this question. (**Q5**) What is their mechanism of membrane permeabilization? **Answer**: There have been few studies in this area, and only for KL peptides have a model been proposed (13), as illustrated in Figure 1. Binding of cationic XZ peptides to anionic membranes is clearly driven by long-range electrostatic attraction, but favorable hydrophobic interactions will also enable zwitterionic vesicles to be covered by peptides to a considerable extent. They then probably act via a “carpet mechanism,” but have not been observed to form transient pores or well-defined β-barrels. Instead, lateral crowding in the outer monolayer of the plasma membrane seems to be responsible for imminent antimicrobial action. At this stage, both peptide length as well as concentration have to be high enough that the molecules assume a β-stranded conformation and become self-assembled. When pre-aggregation into amyloid-like fibrils is too vigorous and occurs already in solution, this material is lost and antimicrobial activity decreases. These pre-aggregated oligomers nonetheless seem to be membranolytic against red blood cells (but not bacteria), so hemolysis becomes more and more pronounced the longer and more aggregation-prone the peptides are.

Since the therapeutic potential of a peptide can be assessed by comparing MIC and HC_50_, we can conclude that the simple XZ peptides with 6–11 amino acids bear quite some promise. Still, the interest in optimizing these kinds of peptides seems to be low. So far, no systematic effort has been made to compare all suggested combinations of cationic and hydrophobic amino acids, and in most studies only a few discrete peptides lengths have been used. There is still much work needed to get a clear picture of these simple peptides, to determine which sequence and length has the most promising therapeutic potential, to resolve the remaining mechanistic details in each particular case. There is also a need of studies of peptide stability, and to determine if microbes develop resistance and if peptides are effective *in vivo*.

## Author Contributions

All authors listed have made a substantial, direct and intellectual contribution to the work, and approved it for publication.

## Conflict of Interest

The authors declare that the research was conducted in the absence of any commercial or financial relationships that could be construed as a potential conflict of interest.
