# Supplementary material for: Antibiotic Potential and Biophysical Characterization of Amphipathic β-Stranded [XZ]n Peptides With Alternating Cationic and Hydrophobic Residues
Source: Front Med Technol. 2021 Feb 4;3:622096. doi: 10.3389/fmedt.2021.622096 (PMC8757834; doi:10.3389/fmedt.2021.622096)
Supplement: Supplementary file 1 [file Data_Sheet_1.pdf]

Antibiotic Potential and Biophysical Characterization of Amphipathic β-Stranded $[XZ]_n$ Peptides With Alternating Cationic and Hydrophobic Residues

Erik Strandberg, Parvesh Wadhwani, and Anne S. Ulrich

Karlsruhe Institute of Technology, Karlsruhe, Germany

Supporting Information

TABLE S1. CD results for $[XZ]_n$ peptides in different environments. (Abbreviations for secondary structures: α, α-helical structures; β, β-like structures; AP β, antiparallel β-sheet; Rc, random coil.)

**Peptide**	**Environment**							**Reference**
	**Method**	**Water**	**PB**	**Buffer**	**SDS micelles**	**Vesicles**		
						Neutral	Anionic	
$[KL]_8$	CD	β						(Baumruk et al. 1994)
$[KL]_{10}$	CD	Rc/β[a]						(Baumruk et al. 1994)
$[KL]_8$	IR	AP β						(Baumruk et al. 1994)
$[KL]_n$	CD	Rc/β[b]						(Brack and Spach 1981)
$[KL]_n$	IR	AP β						(Brack and Spach 1981)
Dansyl-$[KL]_4$K-NH_2	IR	AP β				AP β (DMPC monolayer)		(Castano et al. 2000)
Dansyl-$[KL]_5$K-NH_2	IR	AP β				AP β (DMPC monolayer)		(Castano et al. 2000)
Dansyl-$[KL]_6$K-NH_2	IR	AP β				AP β (DMPC monolayer)		(Castano et al. 2000)
Dansyl-$[KL]_7$K-NH_2	IR	AP β				AP β (DMPC monolayer)		(Castano et al. 2000)
C_{16}-$[VK]_4$	CD	β						(de Almeida et al. 2019)
L$[KL]_3$	CD			β (Tris)				(DeGrado and Lear 1985)
L$[KL]_3$	IR			β (Tris)				(DeGrado and Lear 1985)
$[KW]_3$	CD		Rc			Rc (EYPC/Chol)[e]	Rc+β (EYPE/EYPG)[f]	(Gopal et al. 2011)
$[WK]_3$	CD		Rc			Rc (EYPC/Chol)[e]	Rc+β (EYPE/EYPG)[f]	(Gopal et al. 2011)
$[KL]_4$K	CD	Rc		β (PBS)				(Hernández et al. 2006)
$[KL]_7$K	CD	Rc		β (PBS)				(Hernández et al. 2006)
RW-NH_2	CD		Rc			Rc (POPC in PB)[g]	Rc (POPG in PB)[h]	(Liu et al. 2007)
$[RW]_2$-NH_2	CD		Rc			Rc (POPC in PB)[g]	Rc (POPG in PB)[h]	(Liu et al. 2007)
$[RW]_3$-NH_2	CD		Rc			Rc (POPC in PB)[g]	Rc+β (POPG in PB)[h]	(Liu et al. 2007)
$[RW]_4$-NH_2	CD		Rc			Rc+β (POPC in PB)[g]	Rc+β (POPG in PB)[h]	(Liu et al. 2007)
$[RW]_5$-NH	CD		Rc			β (POPC in PB)[g]	β (POPG in PB)[h]	(Liu et al. 2007)
$[VRVK]_2$ -NH_2	CD	Rc			β			(Ong et al. 2013)
$[IRIR]_2$ -NH_2	CD	Rc			β			(Ong et al. 2013)
$[IKIK]_2$-NH_2	CD	Rc			β			(Ong et al. 2013)
$[IRIK]_2$-NH_2	CD	Rc			β			(Ong et al. 2013)
$[IRVK]_2$-NH_2	CD	Rc			β			(Ong et al. 2013)
$[FRFK]_2$-NH_2	CD	Rc			β			(Ong et al. 2013)
$[WRWK]_2$-NH_2	CD	Rc						(Ong et al. 2013)
$[VRVK]_3$-NH_2	CD	Rc			β			(Ong et al. 2013)
$[IRIK]_3$-NH_2	CD	Rc			β			(Ong et al. 2013)
$[IRVK]_3$-NH_2	CD	Rc			β			(Ong et al. 2013)

Ac-$[KV]_2$-$NHCH_3$	CD			Rc (Hepes)		Rc (DPPC)	Rc (DPPC/DPPG)[d]	(Ono et al. 1990)
Ac-$[KV]_3$-$NHCH_3$	CD			Rc (Hepes)		Rc (DPPC)	α (DPPC/DPPG)[d]	(Ono et al. 1990)
Ac-$[KV]_4$-$NHCH_3$	CD			Rc (Hepes)		Rc (DPPC)	β (DPPC/DPPG)[d]	(Ono et al. 1990)
$[KL]_3$-NH_2	CD	Rc					Rc (POPC/POPG)[c]	(Schweigardt 2020)
$[KL]_4$-NH_2	CD	Rc					β (POPC/POPG)[c]	(Schweigardt 2020)
$[KL]_4$K-NH_2	CD	Rc					β (POPC/POPG)[c]	(Schweigardt 2020)
L$[KL]_4$-NH_2	CD	Rc					β (POPC/POPG)[c]	(Schweigardt 2020)
$[KL]_5$-NH_2	CD	Rc					β (POPC/POPG)[c]	(Schweigardt 2020)
$[LK]_5$-NH_2	CD	Rc					β (POPC/POPG)[c]	(Schweigardt 2020)
$[KL]_5$K-NH_2	CD	Rc					β (POPC/POPG)[c]	(Schweigardt 2020)
L$[KL]_5$-NH_2	CD	Rc					β (POPC/POPG)[c]	(Schweigardt 2020)
$[KL]_6$-NH_2	CD	Rc					β (POPC/POPG)[c]	(Schweigardt 2020)
$[KL]_6$K-NH_2	CD	Rc					β (POPC/POPG)[c]	(Schweigardt 2020)
L$[KL]_6$-NH_2	CD	Rc					β (POPC/POPG)[c]	(Schweigardt 2020)
$[KL]_7$-NH_2	CD	Rc					β (POPC/POPG)[c]	(Schweigardt 2020)
$[KL]_7$K-NH_2	CD	Rc					β (POPC/POPG)[c]	(Schweigardt 2020)
L$[KL]_7$-NH_2	CD	Rc					β (POPC/POPG)[c]	(Schweigardt 2020)
$[KL]_8$-NH_2	CD	Rc					β (POPC/POPG)[c]	(Schweigardt 2020)
$[KL]_9$-NH_2	CD	Rc					β (POPC/POPG)[c]	(Schweigardt 2020)
$[KL]_{10}$-NH_2	CD	Rc					β (POPC/POPG)[c]	(Schweigardt 2020)
$[KL]_{11}$-NH_2	CD	Rc					β (POPC/POPG)[c]	(Schweigardt 2020)
$[KL]_{12}$-NH_2	CD	Rc					β (POPC/POPG)[c]	(Schweigardt 2020)
$[KL]_{13}$-NH_2	CD	Rc					β (POPC/POPG)[c]	(Schweigardt 2020)
$[KL]_3$-NH_2	CD	Rc	Rc			Rc (DMPC)	Rc (POPC/POPG)[c]	(Strandberg et al. 2020)
$[KL]_5$-NH_2	CD	Rc	β			Rc (DMPC)	β (POPC/POPG)[c]	(Strandberg et al. 2020)
$[KL]_7$-NH_2	CD	Rc	β			Rc (DMPC)	β (POPC/POPG)[c]	(Strandberg et al. 2020)
$[KL]_9$-NH_2	CD	Rc	β			Rc (DMPC)	β (POPC/POPG)[c]	(Strandberg et al. 2020)

[a] Random coil at low concentrations up to 3.55 mg/ml, β-structure at high concentrations from 7.1 mg/ml, no salt.

[b] Random coil in pure water, β-structure in 0.2 M NaCl.

[c] POPC/POPG (1/1 mol/mol) in water, pH ≈ 6

[d] DPPC/DPPG (3/1) in 5 mM Hepes buffer, pH 7.4

[e] Egg yolk-PC/cholesterol (10/1 w/w) in 10 mM sodium phosphate buffer

[f] Egg yolk-PE/egg yolk-PG (7/3 w/w) in 10 mM sodium phosphate buffer

[g] POPC in 10 mM phosphate buffer, 100 mM NaCl, pH 7.4. Strong Trp bands makes the secondary structure analysis uncertain. The stated secondary structures are based on our own analysis of the published spectra.

[h] POPG in 10 mM phosphate buffer, 100 mM NaCl, pH 7.4. Strong Trp bands makes the secondary structure analysis uncertain. The stated secondary structures are based on our own analysis of the published spectra.

TABLE S2. Overview of antimicrobial and antifungal assay results on XZ peptides. Exact values of MIC and details about the assay used can be found in the references.

**Microorganism**	**Antimicrobial activity (MIC values)**			**Reference**
	**High (<10 μg/ml)**	**Medium (10-100 μg/ml)**	**Low (>100 μg/ml)**	
**Gram-negative bacteria**				
E. coli DSM 30083		$[RW]_3$, $K[RW]_3$, $[RW]_3K$		(Albada et al. 2012)
A. baumannii DSM 30007		$[RW]_3$	$K[RW]_3$, $[RW]_3K$	(Albada et al. 2012)
P. aeruginosa DSM 50071			$[RW]_3$, $K[RW]_3$, $[RW]_3K$	(Albada et al. 2012)
A. laidlawii A-EF22	$[KL]_4K$	$[KL]_5K$, $[KL]_7K$		(Beven et al. 2003)
E. coli ATCC 25922			Acetyl-$[KL]_9$-NH_2	(Blondelle and Houghten 1992)
P. aeruginosa ATCC 27853			Acetyl-$[KL]_9$-NH_2	(Blondelle and Houghten 1992)
E. coli KCTC 1682		$[KW]_3$	$[WK]_3$	(Gopal et al. 2011)
S. typhimurium KCTC 1926		$[WK]_3$, $[KW]_3$		(Gopal et al. 2011)
P. aeruginosa KCTC 1637		$[WK]_3$, $[KW]_3$		(Gopal et al. 2011)
E. coli D31	$[RW]_4$, $[RW]_5$	$[RW]_3$	RW, $[RW]_2$	(Liu et al. 2007)
E. coli	$[IRIR]_2$	$[IKIK]_2$, $[IRIK]_2$, $[IRVK]_2$, $[FRFK]_2$, $[WRWK]_2$, $[VRVK]_3$, $[IRVK]_3$	$[VRVK]_2$, $[IRIK]_3$	(Ong et al. 2013)
P. aeruginosa	$[IRIR]_2$	$[IKIK]_2$, $[IRIK]_2$, $[IRVK]_2$, $[FRFK]_2$, $[VRVK]_3$, $[IRVK]_3$	$[VRVK]_2$, $[WRWK]_2$, $[IRIK]_3$	(Ong et al. 2013)
E. coli DSM 1116	$[KL]_5$, $[LK]_5$, $[KL]_5K$, $L[KL]_5$, $[KL]_6$, $[KL]_6K$, $L[KL]_6$, $[KL]_7$, $[KL]_7K$,	$[KL]_4$, $[KL]_4K$, $L[KL]_4$, $L[KL]_7$, $[KL]_8$, $[KL]_9$, $[KL]_{10}$, $[KL]_{11}$, $[KL]_{12}$, $[KL]_{13}$	$[KL]_3$	(Schweigardt 2020; Strandberg et al. 2020)
E. helveticus DSM 18390	$[KL]_4K$, $L[KL]_4$, $[KL]_5$, $[LK]_5$, $[KL]_5K$, $L[KL]_5$, $[KL]_6$, $[KL]_6K$, $L[KL]_6$, $[KL]_7$, $[KL]_7K$, $[KL]_8$	$[KL]_4$, $L[KL]_7$, $[KL]_9$, $[KL]_{10}$, $[KL]_{11}$, $[KL]_{12}$, $[KL]_{13}$	$[KL]_3$	(Schweigardt 2020; Strandberg et al. 2020)
E. coli 2592		$[LK]_7$, $[LK]_9$, $[LK]_{12}$, $[LR]_7$	$[LK]_8$, $[LK]_{10}$, $[LK]_{11}$, $[LR]_9$, $[LR]_{11}$	(Strom and Brondsema 2006)
P. aeruginosa 10145		$[LK]_9$, $[LK]_{11}$, $[LK]_{12}$	$[LK]_7$, $[LK]_8$, $[LK]_{10}$, $[LR]_7$, $[LR]_9$, $[LR]_{11}$	(Strom and Brondsema 2006)
E. coli ATCC 25922	$[RW]_3$	$[WR]_3$, $W[RW]_2$	$[RW]_2R$, $[WR]_2$, WRW, RWR	(Strøm et al. 2003; Strøm et al. 2002)
P. aeruginosa ATCC 27853		$[RW]_3$, $W[RW]_2$, $[RW]_2R$		(Strøm et al. 2002)
**Gram-positive bacteria**				
S. aureus DSM 20231		$[RW]_3$, $K[RW]_3$, $[RW]_3K$		(Albada et al. 2012)
S. aureus ATCC 43300	$[RW]_3$, $K[RW]_3$	$[RW]_3K$		(Albada et al. 2012)
B. subtilis 168 DSM 402	$[RW]_3$, $K[RW]_3$, $[RW]_3K$			(Albada et al. 2012)
M. gallisepticum S6	$[KL]_4K$	$[KL]_5K$, $[KL]_7K$		(Beven et al. 2003)

S. aureus ATCC 29213			Acetyl-[KL]$_9$-NH$_2$	(Blondelle and Houghten 1992)
S. aureus KCTC 1621			[WK]$_3$, [KW]$_3$	(Gopal et al. 2011)
B. subtilis KCTC 1918			[WK]$_3$, [KW]$_3$	(Gopal et al. 2011)
L. monocytogenes KCTC 3710		[KW]$_3$	[WK]$_3$	(Gopal et al. 2011)
S. aureus ATCC BAA-44	[RW]$_3$, [RW]$_4$, [RW]$_5$		RW, [RW]$_2$	(Liu et al. 2007)
S. epidermidis	[IRIR]$_2$, [IKIK]$_2$, [IRIK]$_2$, [IRVK]$_2$, [FRFK]$_2$, [WRWK]$_2$, [VRVK]$_3$, [IRIK]$_3$, [IRVK]$_3$	[VRVK]$_2$		(Ong et al. 2013)
S. aureus		[IRIR]$_2$, [IRIK]$_2$, [WRWK]$_2$, [VRVK]$_3$, [IRIK]$_3$, [IRVK]$_3$	[IKIK]$_2$, [VRVK]$_2$, [IRVK]$_2$, [FRFK]$_2$	(Ong et al. 2013)
B. subtilis DSM 347	L[KL]$_4$, [KL]$_5$, [LK]$_5$, [KL]$_5$K, L[KL]$_5$, [KL]$_6$, [KL]$_6$K, L[KL]$_6$, [KL]$_7$, [KL]$_7$K	[KL]$_4$, [KL]$_4$K, L[KL]$_7$, [KL]$_8$, [KL]$_9$, [KL]$_{10}$, [KL]$_{11}$, [KL]$_{12}$, [KL]$_{13}$	[KL]$_3$	(Schweigardt 2020; Strandberg et al. 2020)
S. xylosus DSM 20287	[KL]$_4$, [KL]$_4$K, L[KL]$_4$, [KL]$_5$, [LK]$_5$, [KL]$_5$K, L[KL]$_5$, [KL]$_6$, [KL]$_6$K, L[KL]$_6$, [KL]$_7$, [KL]$_7$K, [KL]$_8$, [KL]$_9$, [KL]$_{12}$	L[KL]$_7$, [KL]$_{10}$, [KL]$_{11}$, [KL]$_{13}$	[KL]$_3$	(Schweigardt 2020; Strandberg et al. 2020)
S. aureus ATCC 25923	[WR]$_3$, [RW]$_3$	W[RW]$_2$, [RW]$_2$R	[WR]$_2$, WRW, RWR	(Strøm et al. 2003; Strøm et al. 2002)
**Fungal strains**				
C. albicans KCTC 7270		[WK]$_3$, [KW]$_3$		(Gopal et al. 2011)
T. beigelli KCTC 7707	[KW]$_3$	[WK]$_3$		(Gopal et al. 2011)
F. solani KCTC 6326	[KW]$_4$, [KW]$_5$, [RW]$_4$, [RW]$_5$	[KW]$_3$, [RW]$_3$	[KW]$_2$, [RW]$_2$	(Gopal et al. 2012)
F. oxysporum KCTC 6076	[KW]$_5$, [RW]$_5$	[KW]$_3$, [KW]$_4$, [RW]$_3$, [RW]$_4$	[KW]$_2$, [RW]$_2$	(Gopal et al. 2012)
C. albicans	[IKIK]$_2$, [IRIK]$_2$, [IRVK]$_2$	[VRVK]$_2$, [IRIR]$_2$, [FRFK]$_2$, [VRVK]$_3$	[WRWK]$_2$, [IRIK]$_3$, [IRVK]$_3$	(Ong et al. 2013)
C. albicans 10231			[LK]$_7$, [LK]$_8$, [LK]$_9$, [LK]$_{10}$, [LK]$_{11}$, [LK]$_{12}$, [LR]$_7$, [LR]$_9$, [LR]$_{11}$	(Strom and Brondsema 2006)
Cr. Neoformans 32045	[LK]$_7$, [LR]$_7$	[LK]$_8$, [LK]$_9$, [LK]$_{10}$, [LK]$_{11}$, [LK]$_{12}$, [LR]$_9$, [LR]$_{11}$		(Strom and Brondsema 2006)
**Resistant strains**				
E. coli CCARM 1229		[KW]$_3$	[WK]$_3$,	(Gopal et al. 2011)
E. coli CCARM 1238			[WK]$_3$, [KW]$_3$	(Gopal et al. 2011)
C. albicans CCARM 14001		[WK]$_3$, [KW]$_3$		(Gopal et al. 2011)
C. albicans CCARM 14007		[WK]$_3$, [KW]$_3$		(Gopal et al. 2011)
C. albicans CCARM 14020		[WK]$_3$, [KW]$_3$		(Gopal et al. 2011)
S. aureus (methillicin resistant) 33591			[LK]$_7$, [LK]$_8$, [LK]$_9$, [LK]$_{10}$, [LK]$_{11}$, [LK]$_{12}$, [LR]$_7$, [LR]$_9$, [LR]$_{11}$	(Strom and Brondsema 2006)

References

Albada HB, Prochnow P, Bobersky S, Langklotz S, Schriek P, Bandow JE, Metzler-Nolte N. Tuning the activity of a short Arg-Trp antimicrobial peptide by lipidation of a C- or N-terminal lysine side-chain. *ACS Med Chem Lett.* (2012) 3:980-984. doi: 10.1021/ml300148v

Baumruk V, Huo DF, Dukor RK, Keiderling TA, Lelievre D, Brack A. Conformational study of sequential Lys and Leu based polymers and oligomers using vibrational and electronic CD spectra. *Biopolymers.* (1994) 34:1115-1121. doi: 10.1002/bip.360340815

Beven L, Castano S, Dufourcq J, Wieslander Å, Wroblewski H. The antibiotic activity of cationic linear amphipathic peptides: lessons from the action of leucine/lysine copolymers on bacteria of the class *Mollicutes*. *Eur J Biochem.* (2003) 270:2207-2217. doi: 10.1046/j.1432-1033.2003.03587.x

Blondelle SE, Houghten RA. Design of model amphipathic peptides having potent antimicrobial activities. *Biochemistry*. (1992) 31:12688-12694. doi: 10.1021/Bi00165a020

Brack A, Spach G. Multiconformational synthetic polypeptides. *J Am Chem Soc.* (1981) 103:6319-6323. doi: 10.1021/ja00411a009

Castano S, Desbat B, Dufourcq J. Ideally amphipathic β-sheeted peptides at interfaces: structure, orientation, affinities for lipids and hemolytic activity of $(KL)_mK$ peptides. *Biochim Biophys Acta.* (2000) 1463:65-80. doi: 10.1016/S0005-2736(99)00175-3

de Almeida NR, Han YC, Perez J, Kirkpatrick S, Wang YL, Sheridan MC. Design, synthesis, and nanostructure-dependent antibacterial activity of cationic peptide amphiphiles. *ACS Appl Mater Inter.* (2019) 11:2790-2801. doi: 10.1021/acsami.8b17808

DeGrado WF, Lear JD. Induction of peptide conformation at apolar/water interfaces. 1. A study with model peptides of defined hydrophobic periodicity. *J Am Chem Soc.* (1985) 107:7684-7689. doi: 10.1021/ja00311a076

Gopal R, Kim YJ, Seo CH, Hahm KS, Park Y. Reversed sequence enhances antimicrobial activity of a synthetic peptide. *J Pept Sci.* (2011) 17:329-334. doi: 10.1002/psc.1369

Gopal R, Na H, Seo CH, Park Y. Antifungal activity of $(KW)_n$ or $(RW)_n$ peptide against *Fusarium solani* and *Fusarium oxysporum*. *Int J Mol Sci.* (2012) 13:15042-15053. doi: 10.3390/ijms131115042

Hernández B, Boukhalfa-Heniche FZ, Seksek O, Coïc YM, Ghomi M. Secondary conformation of short lysine- and leucine-rich peptides assessed by optical spectroscopies: effect of chain length, concentration, solvent, and time. *Biopolymers.* (2006) 81:8-19. doi: 10.1002/bip.20366

Liu ZG, Brady A, Young A, Rasimick B, Chen K, Zhou CH, Kallenbach NR. Length effects in antimicrobial peptides of the $(RW)_n$ series. *Antimicrob Agents Chemother.* (2007) 51:597-603. doi: 10.1128/Aac.00828-06

Ong ZY, Gao SJ, Yang YY. Short synthetic β-sheet forming peptide amphiphiles as broad spectrum antimicrobials with antibiofilm and endotoxin neutralizing capabilities. *Adv Funct Mater.* (2013) 23:3682-3692. doi: 10.1002/adfm.201202850

Ono S, Lee S, Mihara H, Aoyagi H, Kato T, Yamasaki N. Design and synthesis of basic peptides having amphipathic β-structure and their interaction with phospholipid membranes. *Biochim Biophys Acta.* (1990) 1022:237-244. doi: 10.1016/0005-2736(90)90119-9

Schweigardt F (2020) $[KL]_n$ Peptide – Optimierung von β-Faltblatt-Modellpeptiden zur Generierung neuer antimikrobieller Peptide mit therapeutischem Potenzial, PhD Thesis. Karlsruhe Institute of Technology, Karlsruhe, Germany

Strandberg E, Schweigardt F, Wadhwani P, Bürck J, Reichert J, Cravo HLP, Burger L, Ulrich AS. Phosphate-dependent aggregation of $[KL]_n$ peptides affects their membranolytic activity. *Sci Rep.* (2020) 10:12300. doi: 10.1038/s41598-020-69162-0
Strøm MB, Haug BE, Skar ML, Stensen W, Stiberg T, Svendsen JS. The pharmacophore of short cationic antibacterial peptides. *J Med Chem.* (2003) 46:1567-1570. doi: 10.1021/Jm0340039
Strøm MB, Rekdal Ø, Svendsen JS. Antimicrobial activity of short arginine- and tryptophan-rich peptides. *J Pept Sci.* (2002) 8:431-437. doi: 10.1002/Psc.398
Strom RM, Brondsema PJ (2006) Periodic antimicrobial peptides. U.S. Patent No 7091185 Washington, DC: US Patent and Trademark Office.
